# Telomeres and Cancer

**DOI:** 10.3390/life11121405

**Published:** 2021-12-16

**Authors:** Hueng-Chuen Fan, Fung-Wei Chang, Jeng-Dau Tsai, Kao-Min Lin, Chuan-Mu Chen, Shinn-Zong Lin, Ching-Ann Liu, Horng-Jyh Harn

**Affiliations:** 1 Department of Pediatrics, Tungs’ Taichung Metroharbor Hospital, Wuchi, Taichung 435, Taiwan; fanhuengchuen@yahoo.com.tw; 2 Department of Medical Research, Tungs’ Taichung Metroharbor Hospital, Wuchi, Taichung 435, Taiwan; 3 Department of Rehabilitation, Jen-Teh Junior College of Medicine, Nursing and Management, Miaoli 356, Taiwan; chchen1@dragon.nchu.edu.tw; 4 Department of Life Sciences, Agricultural Biotechnology Center, National Chung Hsing University, Taichung 402, Taiwan; 5 Department of Obstetrics & Gynecology, Tri-Service General Hospital, National Defense Medical Center, Taipei 11490, Taiwan; doc30666@gmail.com; 6 School of Medicine, Chung Shan Medical University, Taichung 402, Taiwan; Fernand.tsai@msa.hinet.net; 7 Department of Pediatrics, Chung Shan Medical University Hospital, Taichung 402, Taiwan; 8 Department of Functional Neurosurgery, Xiamen Humanity Hospital, Fujian Medical University, Xiamen 361015, China; horuslin@yahoo.com.tw; 9 The iEGG and Animal Biotechnology Center, and Rong Hsing Research Center for Translational Medicine, National Chung Hsing University, Taichung 402, Taiwan; 10 Buddhist Tzu Chi Bioinnovation Center, Tzu Chi Foundation, Hualien 970, Taiwan; shinnzong@yahoo.com.tw (S.-Z.L.); sagianne@gmail.com (C.-A.L.); 11 Bioinnovation Center, Buddhist Tzu Chi Medical Foundation, Hualien 970, Taiwan; 12 Department of Neurosurgery, Buddhist Tzu Chi General Hospital, Hualien 970, Taiwan; 13 Department of Medical Research, Hualien Tzu Chi Hospital, Hualien 970, Taiwan; 14 Department of Pathology, Buddhist Tzu Chi General Hospital and Tzu Chi University, Hualien 970, Taiwan

**Keywords:** telomerase, telomerase reverse transcriptase, shelterin, CST, promoter mutations

## Abstract

Telomeres cap the ends of eukaryotic chromosomes and are indispensable chromatin structures for genome protection and replication. Telomere length maintenance has been attributed to several functional modulators, including telomerase, the shelterin complex, and the CST complex, synergizing with DNA replication, repair, and the RNA metabolism pathway components. As dysfunctional telomere maintenance and telomerase activation are associated with several human diseases, including cancer, the molecular mechanisms behind telomere length regulation and protection need particular emphasis. Cancer cells exhibit telomerase activation, enabling replicative immortality. Telomerase reverse transcriptase (TERT) activation is involved in cancer development through diverse activities other than mediating telomere elongation. This review describes the telomere functions, the role of functional modulators, the implications in cancer development, and the future therapeutic opportunities.

## 1. Introduction

Cancer is notorious as it can attack any part of the body, rapidly grow beyond its usual boundaries, invade adjoining tissues, and spread to other organs, resulting in uncontrolled proliferation and eventually death. Nearly 10 million deaths were reported in 2020 from cancer, and the risk of getting cancer in a lifetime (before the age of 75 years) is 20% [1]. The most common newly-diagnosed cancers reported worldwide include breast (2.26 million), lung (2.21 million), and colorectal (1.98 million) cancers [1]. Approximately half of the newly diagnosed lung (57%) and pancreatic (52%) cancer cases in the United States are at an advanced or metastatic stage, and the majority of these patients with an early diagnosis of the disease eventually develop tumor progression [2]. The 5-year relative survival rates for advanced-stage cancers, such as lung, colorectal, liver, and pancreatic, remain low, ranging from 3% to 14% even after maximal surgical excision, radiation, chemotherapy, and hormone, immune, and targeted therapies [2]. Thus, none of the standard cancer treatments can completely cure patients at an advanced stage of the disease. Knowledge of the molecular mechanisms influencing tumor growth and invasiveness may lead to novel and effective therapies for the poor prognosis of late-stage cancers.

Cancer formation and progression is a genetic phenomenon with normal cells accruing genomic instability and thereby acquiring the ability to replicate indefinitely, which is the phenotype of immortality [3]. Telomerase, the immortality enzyme, is ubiquitous in all mammalian embryonic tissue and remains active in germs cells but is down-regulated in most somatic tissues [4]. As telomerase activity determines cellular proliferation, it must be tightly regulated to prevent the induction of carcinogenesis [5]. Telomerase reverse transcriptase (TERT), the catalytic subunit responsible for enzyme activity in telomerase, is the rate-limiting factor of human telomerase enzyme activity [5]. Two of the critical telomere-specific proteins involved in the regulation and maintenance of the telomere length are the shelterin and CST complexes [6]. Mutations in the genes encoding these complexes can result in cancers. Thus, understanding the molecular mechanisms of these proteins is fundamental given the therapeutic strategies to manage such diseases.

## 2. Telomeres, a Genetic Time Bomb or a Biological Clock

Human telomeres comprise a hexameric nucleotide repeat sequence (TTAGGG) that is initially double-stranded DNA (dsDNA) but ends with a single-stranded DNA (ssDNA) overhang (G’-overhang). The extended 5′ to 3′ strand contains the G-rich telomeric repeats and is referred to as the G-strand, while the 3′ to 5′ strand is defined as the C-strand [7,8]. During the cell division cycle, the eukaryotic DNA polymerase is unable to completely replicate the sequences at the chromosomal ends. This is because RNA primers attach at the lagging strand during the synthesis of Okazaki fragments, and the resulting shedding RNA leads to telomere shortening [9]. The so-called “end replication problem” results in eventual apoptosis, cellular senescence, and cell cycle arrest [10,11]. Additionally, chromosomes lacking the “capping structure” tend to get truncated and fused with other chromosomes [12]. As such, telomeres are also considered a genetic time bomb or a biological clock for cellular aging [13].

As approximately 50–200 bases are lost from the terminal sequence of chromosomes each time a cell divides [14], and more than a couple of trillion telomere sequences are in the human genome, the spatiotemporal expression of telomerase must be tightly regulated in humans. Apart from the shedding RNA and the generation of the 3′ overhang by the sequence-specific exonuclease activity to resect back the 5′end of telomeres [15], telomere shortenings can occur, irrespective of cell replication, due to accumulative oxidative stress [16], host age [17], gender [18], sex hormones [19], and lifestyle factors, such as the lack or presence of exercise [20], obesity and weight loss [21], smoking [22], and unhealthy diets [23]. However, short telomeres not only result in genomic loss [24,25], shorter lifespan [26,27] and contribute to diseases such as coronary heart disease [28], heart failure [29], osteoporosis [30], diabetes [31], but can also result in genomic instability and elevated telomerase activity, leading to a potential cancer predisposition factor [32]. Hence, proper telomere maintenance is critical for human life.

## 3. The Shelterin Complex

**Figure 1:**
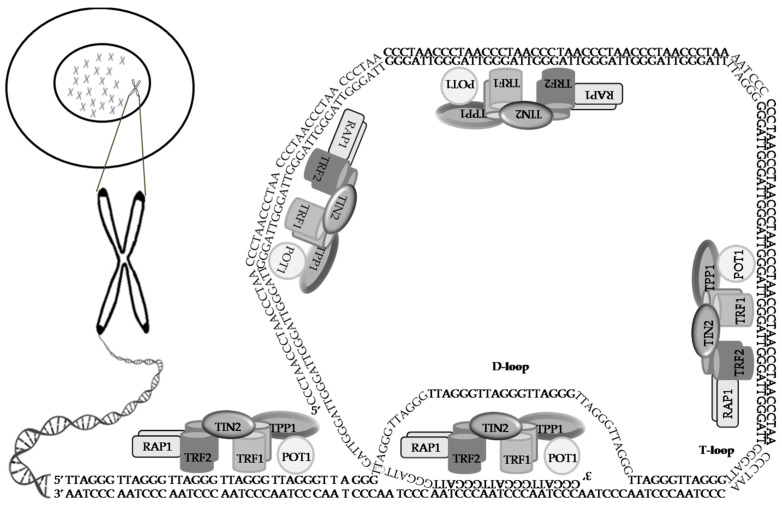
A graphic presentation of telomeric DNA and the proteins that form the shelterin complex. Telomeres are capping structures and are situated at the ends of linear chromosomes. Telomeric DNA, TTAGGG at the chromosome ends, and the complementary DNA strand sequence AATCCC form an extended region of dsDNA ending with a ssDNA G-rich overhang. The 3′ G-rich overhang enables telomeric DNA to form a secondary structure in which the 3′ single-stranded overhang folds back and displaces a strand in the homologous dsDNA TTAGGG region, to create a D-loop that protects the 3′-end from being identified as damaged DNA, thereby preventing the activation of the ataxia-telangiectasia mutation and Rad3-related (ATM/ATR) damage response pathways. The shelterin complex comprises six telomeric proteins: TRF1, TRF2, RAP1, TIN2, POT1, and TPP1. The complex enables the telomeric 3′-overhang/G-tail to fold into a lasso-like structure with a telomeric loop (T-loop) that protects the 3′-end from being recognized for DNA damage and blocks the DNA damage response.

Telomeres are protected by a highly conserved mammalian nucleoprotein complex called shelterin (telosome). This nucleoprotein complex can minimize telomere fragility by enabling DNA replication at the telomeric repeats [33,34]. The shelterin complex can allow DNA to form a lasso-like structure with a telomeric loop (T-loop) and a displacement loop (D-loop) that then shields the 3′-end from DNA damage and blocks the activation of the DNA repair mechanisms, such as ataxia-telangiectasia Rad3-related (ATR)-mediated DNA damage kinase signaling and ataxia-telangiectasia mutation (ATM) kinase cascades, as well as unwanted repair reactions [35]. The shelterin complex anchors to both ssDNA and dsDNA [33,36,37,38]. The shelterin complex and other protein complexes specific to the telomere can detect and react to changes in telomere length in order to maintain the proper length of telomeres [39,40] (Figure 1). However, in cancers, mutations in the shelterin complex that cause telomere dysfunction and dysregulation are very common [41].

The shelterin complex is composed of six subunits, namely the telomere repeat-binding factor 1(TRF1), telomere repeat-binding factor 2 (TRF2), repressor activator protein 1(RAP1), TRF1-interacting nuclear factor 2(TIN2), TINT1/PTOP/PIP1(TPP1), and the protection of telomeres-1(POT1) [42,43].

### 3.1. TRF1

TRF1 is responsible for controlling telomeric DNA extension (Figure 1) [44]. While the over-expression of TRF1 decreases telomere length, dominant-negative TRF1 increases its length, thus suggesting that TRF1 negatively regulates the length of telomeric repeats [45]. In contrast, Karlseder et al. [46] disputed the role of TRF1 in regulating telomere length because they found that complete TRF1 deletion in mice showed no defects in the telomere length or telomere capping.TRF1 is overexpressed in several cancer types, such as renal cell carcinoma [47] and gastrointestinal tumors [48], and is upregulated in glioblastoma multiforme (GBM) [49]. Brain-specific *TRF1* genetic deletion in GBM mouse models inhibited tumor initiation and progression and increased survival. In addition, deleted *TRF1* increased telomeric DNA damage and reduced proliferation and stemness. TRF1 chemical inhibitors mimicked these effects in human GBM cells and also blocked tumor sphere formation [49], suggesting that TRF1 may be a promising target for developing an effective anti-cancer therapeutic strategy.

### 3.2. TRF2

Although TRF2 and TRF1 have similar features, they exhibit distinct functions. The binding affinity of TRF1 to TIN2 is 20-fold greater than that ofTRF2 [50]. TRF2 and TRF1 are both homodimers that can attach to telomeric dsDNA (Figure 1) [51,52,53]. TRF2 acts as a protein hub interacting with many DNA repair proteins. These proteins do contribute to human chromosomal instability syndromes typified by increased cancer and premature aging [33,54]. As a hub protein, TRF2 interacts with multiple proteins, including a specific endonuclease, the Mre11/Nbs1/Rad50 (MNR) complex, poly(ADP-ribose) polymerase 1 (PARP1) and poly(ADP-ribose) polymerase 2 (PARP2), the DNA-protein kinase complex, RAP1, the Bloom syndrome protein (BLM), and the Werner syndrome protein(WRN) helicases [55,56,57,58]. Bloom syndrome (BS) is an autosomal recessive disorder characterized by short stature, a skin rash that develops after exposure to the sun, intellectual disability, microcephaly, and increased chromosome breakage and an increased risk for cancer [59]. Hand and German [60], using diploid fibroblast cell lines derived from skin biopsies taken from five BS patients, observed a slower rate of replication fork movement compared to the normal adult controls. It is now clear that this aberration results from defective RecQ helicase function during DNA replication [61]. Loss-of-function mutations of *BLM*, which codes for a RecQ helicase, cause BS [62]. Patients with Werner syndrome (WS) manifest growth retardation, short stature, premature graying of hair, alopecia, wrinkling, prematurely aged faces with beaked noses, premature brain atrophy, lipodystrophy, gonad atrophy, bilateral cataracts, premature arteriosclerosis, calcinosis, type 2 diabetes, osteoporosis, telangiectasia, and malignancies [63,64]. WS is caused by mutations in the *WRN* gene [65]. WRN, which is coded by the *WRN* gene, is a DNA helicase which maintains genomic stability by participating in double-strand break (DSB) repair and inter-strand crosslink repair, as well as other DNA processing events [66].

TRF2 is also associated with the integrity of the G-strand overhang and the protection of telomeres, as it has been demonstrated that the over-expression of dominant-negative TRF2 in cells results in the loss of both the TRF2 bound at the telomeres and the G-strand overhang, the activation of the p53 damage pathway, and chromosome end fusions [53,67,68]. In addition, TRF2 is essential for T-loop assembly and maintenance of the ATM-mediated DNA damage response (DDR) suppression and repression from non-homologous end joining (NHEJ) [69]. As with the TRF1-knockout mice, TRF2-knockout mice are embryonically lethal [70]. Whereas TRF2 over-expressing mice under the 5′-regulatory region of the keratin 5 (K5) gene have increased vulnerability to spontaneous skin tumors and are sensitive to UV-induced carcinogenesis [71,72,73]. TRF2 production is often raised in human skin carcinomas [71]. Patients with xeroderma pigmentosum who have XPF, a nuclease associated with UV-damage repair, specific mutations are susceptible to TRF2-associated telomere shortening and chromosomal instability [71]. Furthermore, a lack of telomerase significantly accelerates TRF2-induced epithelial carcinogenesis, suggesting a greater chromosomal instability and an increased burden of DNA damage [74], thereby alluding to a role of TRF2 in directing telomere recombination. Similar to TRF1, some studies have reported that TRF2 is up-regulated in some human cancers, such as skin cancers, and increased TRF2 expression can promote skin tumorigenesis [54,74,75]. Bejarano et al. [76] identified a direct link between TRF1 phosphorylation by common cancer signaling pathways, telomere protection, and cancer treatment. Interestingly, the link is not only restricted to TRF1, but also to TRF2 because TRF2 is reported to be phosphorylated by the ERK1/2 kinases and interacts with Ras signaling to bypass DDR in cancer cells [77,78]. Taken together, these findings indicate that targeting common cancer signaling pathways through down-regulating the shelterin complex may be a potential anti-cancer therapeutic strategy.

### 3.3. RAP1

In humans, RAP1 associates with TRF2 and is enlisted to telomeres to control telomere length (Figure 1) [79]. RAP1 improves the selective binding of TRF2 to telomeric DNA [80]. Mammalian RAP1 can shield telomeres from NHEJ activities in vitro and the context of severe telomere uncapping induced by TRF2 dysfunction [81]. Loss of RAP1 function in human cells does not result in NHEJ, homology-directed repair (HDR), or a DNA damage response [82]. At the same time, mouse RAP1 has been shown to shield telomere ends by repressing HDR and preventing sister telomere recombinations [82]. RAP1 binds to extra-telomeric DNA and acts as a transcriptional regulator [82,83]. RAP1 has also been reported to associate with I kappa B (IκB) kinases and activate nuclear factor kappa B (NF-κB) [84]. The expression of RAP1 has been shown to be significantly higher in breast tumor tissues than in the adjacent non-tumor tissues [84], suggesting that RAP1 could be involved in cancer progression. Moreover, RAP1 is reported to be highly expressed in colorectal cancer tissues, and the expression levels of RAP1 are significantly correlated with poor prognosis and metastasis [85].

### 3.4. TIN2

TIN2 directly connects TRF1, TRF2, and TPP1 without binding POT1 or RAP1 (Figure 1) [37,86]. TIN2 regulates the telomere length [38], maintains T-loop [87], and complexes with Rap1 and TPP1 to allow cells to distinguish telomeres from sites of DNA damage [33]. The over-expression of TIN2 has been shown to prevent telomere elongation in several human cell lines, such asHT1080 (human fibrosarcoma cell line), WI-38 (fibroblast-like fetal lung cell line), U2OS (bone osteosarcoma epithelial cell line), HTB9 (bladder carcinoma cell line), C33A (cervical carcinoma cell line), MDA-452 (breast cancer cell line), and HMT-3522 (non-tumorigenic human breast epithelial cell line). In contrast, the expression of a dominant-negative TIN2 results in uncontrolled telomere elongation [88]. Chiang et al. [89] have observed that early embryonic lethality occurs in mice with TIN2 deletion, similar to the findings of TRF1- and TRF2-deficient mice [46,70]. Expression of TRF1, TRF2, and TIN2 have been detected in human gastric carcinoma [48], and down-regulation of TRF1, TRF2, and TIN2 gene expression may be vital to maintain telomeres in gastric cancers [90]. Expressions of TRF1, TRF2, and TIN2 have also been found in patients with adult T-cell leukemia [91]. Increased expression of TRF1, TRF2, and TIN2 is linked to telomere shortening during multi-step hepatocarcinogenesis [92].

### 3.5. TPP1

**Figure 2:**
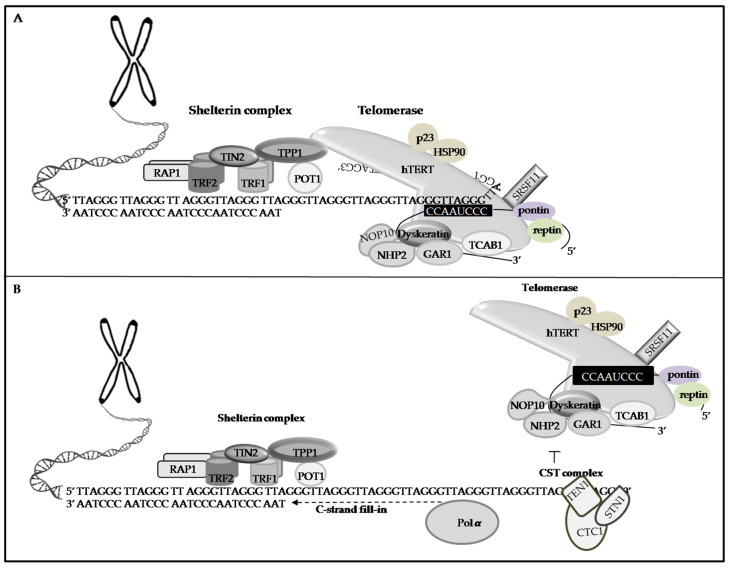
Interaction of the shelterin complex, CST complex and telomerase to maintain telomere length. (**A**) The shelterin complex is essential for telomere protection and for regulating telomere elongation. TIN2, RAP1, and TRF1/2 subunits of the shelterin complex associate with telomeric dsDNA, while POT1 and TPP1 bind telomeric ssDNA and are responsible for recruiting telomerase to the telomeres. The shelterin complex also stimulates extension of the G-overhang by telomerase. (**B**) The CST complex prevents telomerase from engaging the G-overhang and facilitates the C-strand fill-in. The CST complex has three components—conserved telomere protection component 1 (CTC1), suppressor of cdc thirteen 1 (STN1) and telomeric pathway with STN1 (TEN1)—which are thought to function in part in telomere lagging-strand synthesis. The human telomerase consists of the hTERT, the TERC, and accessory proteins. The hTERT can wrap the chromosome to add single-stranded telomere repeats. The TERC contains the template for telomere replication. When an ongoing extension of a stranded DNA is finished, telomerase activity is terminated at the ssDNA overhang by the CST complex, which also activates the C-strand fill-in: the CST complex recruits DNA polymerase alpha (Polα) for lagging strand synthesis of the telomeric C-strand to convert the newly synthesized G-overhang into double-stranded DNA.

TPP1 is a protein associated with POT1 and TPP1-POT1 heterodimer binds and caps the telomeric 3′ tail to protect telomeres (Figure 2A) [93,94]. TPP1-POT1 also binds to internal sites of telomeric ssDNA [95]. TPP1 deletion results in a strong ATR-dependent DDR at telomeres, causing excess telomeric ssDNA [95,96,97]. In addition to telomere protection and maintenance of telomere integrity, TPP1 can recruit telomerase, stabilize its association with the overhang, and stimulate enzyme activity to lengthen the telomeres (Figure 2A) [98]. Furthermore, TPP1 has been reported to promote telomerase processivity in the presence of POT1 [99]. TPP1 loss causes decreased expressionof POT1 at the telomeres and reduced telomerase processing [97,100]. TPP1 interacts with the CST complex and regulates telomere elongation by telomerase [99]. Tejera et al. have found that TPP1 deletion can decrease TERT binding to the telomeres [101]. Interestingly, they have also showed that abrogation of TPP1 abolishe snet telomere elongation in the context of the nuclear reprogramming of TRF1-deficient mouse embryonic fibroblasts into induced pluripotent stem cells (iPSCs), suggesting that TPP1 has a dual role in telomere protection as well as elongation. Another report shows that the complete TPP1 abrogation results in embryonic lethality [102]. Together, TPP1 deficiency can cause telomere dysfunction phenotypes, including widespread epithelial dysplasia, defective hair follicle morphogenesis, growth, severe skin hyperpigmentation, and peri-natal death [103,104,105].

### 3.6. POT1

POT1, like TRF2, plays a major role in end-capping.POT1negatively regulates telomerase and helps in telomere maintenance. POT1 interacts with TPP1 and attaches to the ssDNA 3′ overhang (Figure 1), thereby repressing ATR-mediated DDR by stopping the recruitment of replication protein A (RPA) to the ssDNA [106]. A decrease in POT1 can result in an irreparable ATR pathway with significantly elongated telomeres and cell cycle arrest and is embryonically lethal [93,107,108,109]. Two POT1 orthologs, POT1a and POT1b, are present in the mouse. POT1a is necessary to fully repress a DNA damage signal at telomeres [108]. POT1b has a specific role in regulating the structure of the telomere terminus, leading to deregulation of the telomeric overhang in POT1b-deficient cells, despite the presence of POT1a [108]. POT1b regulates the amount of ssDNA at the telomeres in a telomerase-independent manner [108]. Double knockout cells for the POT1a and POT1b genes have been demonstrated to result in telomere elongation, an increase in DNA damage foci at the telomeres, endo-reduplication, and early initiation of senescence [108,109]. POT1 mRNA concentrations have been significantly linked with telomere length in colon and gastric cancer cells [108]. A known variant of POT1, with D224N mutation, disrupts POT1 binding to ssDNA telomere oligonucleotides, leading to longer and fragile telomeres, predisposing for chronic leukocyte leukemia, glioma, angiosarcoma, osteosarcoma, thyroid cancer, colorectal cancer, and cutaneous melanoma [110]. Convergently, the shelterin complex interacts with more than 300 proteins, including RING-finger- or U-box-containing proteins, functioning as ubiquitin E3 ligases or stability regulators for telomere-associated proteins, protein phosphatase catalytic and regulatory subunits (PPM1G, PHPT1, PTPN5, SAPS3, and PPP1R2), and phosphorylation-related kinases (Akt1, CAMK1D, CLK3, MAP2K3, MAP4K2, MAPK12, and PAK4) [111], indicating that the complex not only stabilizes the chromosomal ends and protects hosts from diseases, but also acts as a busy hub for the complex signaling net workflow.

## 4. The CST Complex

The CST complex comprises telomere-specific proteins that regulate telomere length replication and maintenance. The CST complex was initially identified in Saccharomyces cerevisiae and later in vertebrates [101,112,113].

### 4.1. Yeast CST Complex

Saccharomyces CST complexisa trimeric nucleoprotein complex composed of cell division control protein 13(CDC13), suppressor of CDC thirteen 1 (STN1), and telomeric pathway with STN1 (TEN1) [114]. During cell budding, the CST complex is known as the CDC13-STN1-TEN1 complex; however, fission yeast only contains STN1 and TEN1 [115]. Deletions affecting CDC13, STN1,or TEN1 make budding yeast cells unviable [114]. Therefore, the yeast CST complex is crucially important and may possess evolutionarily conserved functions in DNA replication [116]. Yeast CST complex is structurally related to the heterotrimeric replication protein A (RPA)-complex [112], which is a heterotrimeric ssDNA-binding protein complex composed of replication factor A1 (Rfa1), Rfa2, and Rfa3 (Figure 2A) [117].

### 4.2. Human CST Complex

**Figure 3:**
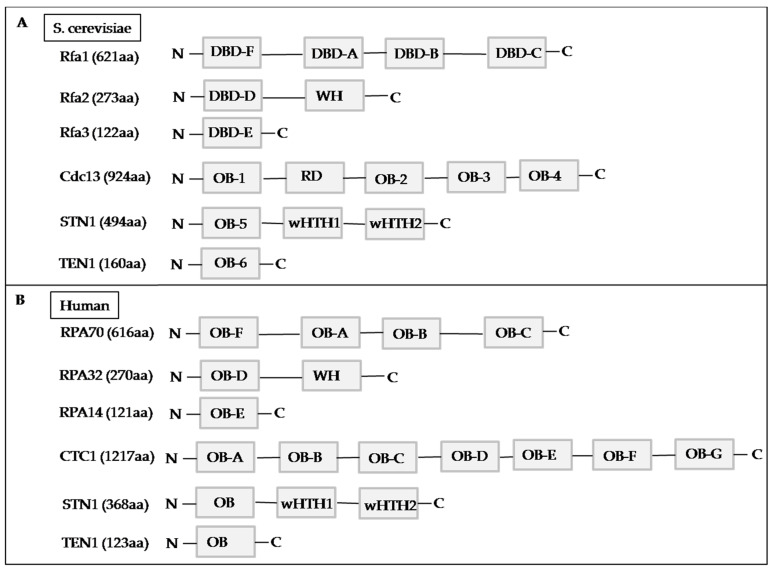
Comparison of (**A**) *S. cerevisiae* Rfa and CST with (**B**) human RPA and CST. Domain structures of Rfa and CST. DBD: DNA-binding domain;OB: OB-fold domain; RD: recruitment domain; TR2: the single RAD51-binding domain; WH: winged helix domain; wHTH: winged helix-turn-helix domain.

As with yeast, the human CST complex comprises the conserved telomere maintenance component 1 (CTC1), STN1, and TEN1 (Figure 2B), and each subunit is present in the stoichiometric ratio of 1:1:1 [118,119,120]. It localizes at the chromosomal ends, preferentially to G-rich and repetitive elements [121], and can maintain telomere length [113,122]. Human CST is an RPA-like ssDNA-binding protein that has primarily been characterized as a telomere replication factor [123]. RPA is crucial for replication, repair, and recombination and is involved in multiple protein–protein interactions [124], telomere metabolism [112], and chromosome maintenance [125]. The human RPA complex comprises RPA70, RPA32, and RPA14 [117,126]. Structural analyses have demonstrated that each RPA subunit contains multiple oligonucleotide/oligosaccharide-binding domain (OB) (four in Rfa1/RPA70, one in Rfa2/RPA32, and one in Rfa3/RPA14) [126]. In yeast, the OB folds are DNA binding domains (DBD) and protein-protein interaction domains [127]. Rfa1,the largest subunit, has four OB domains: DBD-A, DBD-B, DBD-C, and DBD-F, connected by flexible linkers. Rfa2 has one OB fold (DBD-D) followed by a winged-helix (WH) domain, which participates in protein-protein interactions [126]. Rfa3, the smallest subunit, possesses one OB fold (DBD-E). Among these OB-fold domains, DBD-A, DBD-B, DBD-C, and DBD-D are the key players in RPA’s ssDNA-binding activities, while DBD-F and DBD-E have weak interactions with DNA [112,128,129]. In humans, the three RPA subunits, includingRPA70, containing four OB-fold domains (OB-A, OB-B, OB-C, and OB-F), RPA32, containing an OB-fold domain (OB-D) and a WH domain, and RPA14, the smallest subunit, containing an OB-fold domain, OB-E, form a trimerization structure. Among these OB-fold domains, the high binding affinity of RPA to ssDNA is mostly facilitated by OB-A, OB-B, OB-C, and OB-D in RPA70 and RPA32, while the OB-F and WH domains interact with its protein-binding partners [126,128,130] (Figure 3B). The OB-fold domains are connected with flexible linkers [131]. Each OB-fold domain is a five-stranded β barrel structural motif, existing in different proteins for nucleic acid recognition [132]. In addition to the recognition of ssDNA, the functions of the OB fold include the location of the binding surface, the polarity of the nucleic acid with respect to the OB fold, recognition of unusually structured nucleic acids, and a sensor of DNA damage [132]. Functionally, CST has several features:1. Binding to ssDNA with G-rich sequences [119,133,134,135,136,137]. 2. Binding to ssDNA-dsDNA junctions [138]. 3. Recognizing different specialized DNA structures at the DNA replication and breakage sites [139]. 4. Acting synergistically with ATR to maintain telomere length and genome stability [140]. 5. Stimulating the primase activity of DNA polymerase alpha (Polα) switch from RNA to DNA synthesis [141,142,143]. 6. Helping in C-strand fill-in (Figure 3B) [136,144].7. Preventing the accumulation of G4: CST can directly engage and melt G4 DNA structures and prevent the accumulation of G4 structures during unperturbed DNA replication [139]. 8. Preventing telomeric DNA damage: CST can speed up telomeric DNA replication by facilitating the restart of the stalled replication forks to prevent telomeric DNA damage [125,134]. 9. Interacting with the mini chromosome maintenance of the 2–7(MCM) complex and disrupting the binding of CDT1 to MCM, leading to decreased origin licensing [145]. 10. Interacting with acidic and nucleoplasmic DNA-binding protein (AND-1), which is an important regulator governing the assembly of the MCM complex at the replication origins during the origin licensing in human cells [146]. 11. Inhibiting telomerase (Figure 2B): The CST complex interacts with shelterin or telomeric ssDNA to terminate telomerase for telomere length homeostasis, and this reaction does not need TEN1 [138,143,144,147,148]. In addition, CST interacts with and sequesters telomeric 3′ overhangs after their elongation by telomerase, thus preventing telomere extension by telomerase [120]. Therefore, impaired CST’s functions may increase the possibilities of defective telomere replication, deregulation of telomere length, and replication fork stalling, leading to irreparable DNA damage, genome instability, and diseases [125,136] (Table 1).

Similar to CST, RPA has several functional features:1. Binding ssDNA: RPA binds and protects ssDNA from cleavage by nucleases and recruits repair proteins to initiate DNA damage responses [149]. 2. Activating the ATR signaling: Replication stress (RS) is a condition when the replication fork progression and/or DNA synthesis is stalled or slowed [150]. The RPA-coated ssDNA serves as a main activation platform for recruiting ATR-ATR-interacting protein (ATRIP) to the stalled forkin RS [151]. Activated ATR-ATRIP phosphorylates and activates Checkpoint kinase 1(CHK1), which induces cell cycle arrest to allow DNA repair, fork stabilization, or replication start [156]. 3: Activating the helicase: RPA binding stimulates the accumulation of the human DNA helicase B on chromatin in replication stress [157].4. Unwinding G4: RPA binding promotes WRN activity and multiple RPA binding makes WRN a super-helicase on G4 unwinding [152]. 5. Involvement in DNA replication, recombination, and repair: BLM forms a complex with topoisomerase IIIα, RPA, and several factors involved in functions related to DNA replication, recombination, and repair [62]. 6. Activating BLM’s bidirectional DNA unwinding [153]. 7. Modulating the fork remodeling enzyme activity: SMARCAL-1(SWI/SNF-related, matrix-associated, actin-dependent regulator of chromatin, subfamily A-like 1) is a fork-remodeling enzyme. RPA binds to ssDNA at the fork junction, creating an optimal DNA-protein substrate for SMARCAL-1. However, when the RPA binding to the ssDNA formed at the leading strand stimulates the SMARCAL1-mediated fork remodeling activity, the RPA binding at the lagging strand inhibits the SMARCAL1 activity [154]. 8. Enhancing primase: RPA enhances primase activity at forks [155] (Table 1).

### 4.3. CTC1

CTC1 and CDC13 are the largest subunits of the human and budding yeast CST complexes, respectively. As the CDC13 null strain cannot be generated in yeast cells [158], it is believed that CDC13 is essential for cell viability. Although STN1 and TEN1 are highly conserved [159], the genomic sequences and functions of CDC13 in yeast and that of CTC1 in humans are different [120]. Structurally, CDC13 consists of OB1, OB2, OB3, and OB4 (Figure 3A). The roles of these OBs include ssDNA binding, protein–protein interactions, DNA polymerase α-primase binding, and CDC13 homo-dimerization [160,161,162]. However, a recent study analyzing the crystal structures of the CST complex of *Kluyveromyces lactis* suggests that OB2 and OB4 are required for the CDC13–STN1 interaction that assembles CST in a 2:2:2, instead of1:1:1, stoichiometry [163].

Human CTC1 has OB-A, OB-B, OB-C, OB-D, OB-E, OB-F, and OB-G (Figure 3B). The C terminus of CTC1 (OB-D through OB-G) acts as a platform to assemble STN1 and TEN1 [118]. STN1 (STN1 OB and the first winged helix-turn-helix [wHTH1] domain of STN1) interacts with CTC1 at two interaction sites, CTC1 OB-G and CTC1 OB-E, respectively (Figure 3B) [118]. Structural analyses have shown that CTC1 OB-G is similar to the OB-C of RPA70 and not CDC13 [128]. The CDC13 recruitment domain (RD) contains numerous phosphorylation sites [164,165,166,167,168]. Phosphorylated CDC13 RD enhances the ever shorter telomere 1 (Est1), a component of the yeast telomerase holoenzyme binding and telomerase recruitment to telomeres [164,165]. Est1 is present in humans and a report shows that the expression of Est1 is significantly reduced in B-chronic lymphocytic leukemia [169]. Dephosphorylated CDC13 RD promotes CST complex assembly to bind and cap the ends of chromosomes [165,166,167,170]. Human CTC1 represses the elongation of telomerase by binding to telomerase-extended telomeres thus preventing telomerase activity [144]. CDC13–Est1 and POT–TPP1 are essential in directing telomerase to the chromosomal ends [171,172]. CTC1 interacts with TPP1 to compete with TPP1–POT1 for binding at the telomeric 3′ tail and sequestrate the single-stranded telomeric overhang to inhibit the telomerase extension reaction [120,144]. In humans and yeast, the CST complex prevents 3′ overhangs via boosting the fill-in synthesis [148,173]. Yeast CDC13 deficiency causes genome stability and unstable chromosomes [174]. Dyskeratosis congenita (DC) and Coats plus syndrome (CPS) are two uncommon diseases associated with mutations that affect the CST complex. CPS is an autosomal recessive, systemic disorder characterized by intrauterine growth retardation, bilateral exudative retinal telangiectasias, intracranial calcifications, intracerebral cysts, extra-neurological features, including osteopenia with a tendency of fractures and gastrointestinal bleeding, and portal hypertension [175]. Symptoms of DC include increased cancer incidence, bone marrow failure, lacy reticular pigmentation of the upper chest and/or neck and oral leukoplakia [176]. Changes that occur as a result of CTC1 and STN1 mutations include telomere DNA replication defects, genome instability, defects in interactions with Polα, chromosome breakage, and an accumulation of the ssDNA gaps of telomeric DNA [114,177,178]. Interestingly, some reports have identified shorter telomere length in the lymphocytes of subjects with CTC1 mutations [176,179], but contradictory results were also reported [180,181]. Nevertheless, further studies are crucial to clarify the roles of CST in disease pathogenesis.

### 4.4. STN1

Human STN1 was initially named as Polα accessory factor44 as STN1 has been shown to enhance primase and up-regulate the recruitment of Polα for lagging strand DNA replication [141,182]. Structurally, the yeast STN1 consists of an OB-5 domain and two wHTH motifs, wHTH1 and wHTH2, which may involve Polα and CDC13 binding [118]. The N-terminus of STN1 binds to TEN1, while the C-terminus associates with both CDC13 and Pol12 (the B subunit of Polα) [183,184].The STN1 and TEN1 are enlisted to telomere ends via direct association with CDC13. Both STN1 and TEN1 display relatively poor telomeric DNA-binding affinities [185]. In humans, STN1 functions as an adapter between TEN1 and CTC1 [122], and the STN1 N-terminal interacts with CTC1 OB-G and the C-terminal with CTC1 OB-E [118]. Fluorescence investigation has demonstrated that the STN1-binding sites are prone to DNA breakage in STN1 deficient cells under replication stress, leading to chromosome fragmentation [121]. The human STN1 and TEN1 can associate to form a stable complex in vitro [127,137,159,162,170,171,185,186,187,188], which may be due to the comprehensive interactions between the two C-terminal helices of the OB folds and the contacts between these domain bodies. Additionally, OB-fold functions may be related to its preference for the G-rich sequence [135,189]. CTC1 and STN1 can alone reduce telomerase activity and disrupt telomeric DNA damage signaling [138]. STN1-TEN1 forms a wide nucleic acid binding pocket on the surface of the protein complex [159]. STN1, the shelterin complex, and telomerase may recruit Polα to telomere [190]. A reduction in CTC1 or STN1 produces lengthened G-overhangs as the C-strand fill-in becomes faulty [136,177,191]. STN1mutationlike CTC1 mutation can cause CPS [179,192,193]. Depletion of human CTC1 or STN1 increases multi-telomeric signals, telomere instabilities, and chromosome breakage [191] and can result in impairing C-strand fill-in, leading to excessively long G-overhangs [125,134,136,143]. 

### 4.5. TEN1

Of the CST components, TEN1is the smallest with a single OB fold [118]. Yeast TEN1 may promote the activity of CDC13 and bind to telomeric ssDNA to enhance the DNA-binding activity of CDC13 [194]. TEN1 in humans is to stabilize the binding of CTC1-STN1to ssDNA and to support C-strand fill-in after G-strand extension by telomerase [138]. Human TEN1 attachment to CTC1 OB-G is facilitated by the OB of STN1 [118]. Human TEN1mutant strain proteins are unable promote the binding of CDC13 to telomeres in vitro, indicating that TEN1 improves the telomeric DNA-binding activity of CDC13 that then negatively affects the telomere length [138]. Knockout TEN1 cells show gradual telomere shortening comparable to that resulting from telomerase deficiency [138], indicating that TEN1 is crucial for the maintenance of telomere length. In addition to ensuring telomere stability [119], TEN1 and STN1 can rescue replication fork stalling during replication stress [122,125,195].

CDC13, STN1, and TEN1 are essential for cell viability and regulating telomere length. Subunit mutations resulting in loss-of-function can cause an accumulation of telomeric ssDNA and result in abnormal elongation of the telomeres, indicating that these three subunits are critical to the health of organisms with the CST complex [6,118,133,147]. The interactions between POT1-TPP1 and CST can significantly affect the telomere length and may result in telomere length dysregulation and cancer development, such as familial glioma [196], melanoma [197], chronic lymphocytic leukemia [198] and breast cancers [199,200], stomach cancers [199], and parathyroid cancers [201].

## 5. Telomerase: Breaking through the Limitation of Replication

Telomerase, the enzyme responsible for lengthening the telomeres, can extend the cellular lifespan or induce immortalization [1]. Typically, in healthy adult somatic cells, telomerase is inactive to avoid uncontrolled cellular proliferation [2], whereas in approximately 90% of human tumors, telomerase is up-regulated or reactivated to help tumor cells survive and multiply [202]. However, developing embryos, reproductive cells, activated immune cells, bone marrow, and adult stem cells show high telomerase activity [18].

### 5.1. Components of Telomerase

**Figure 4:**
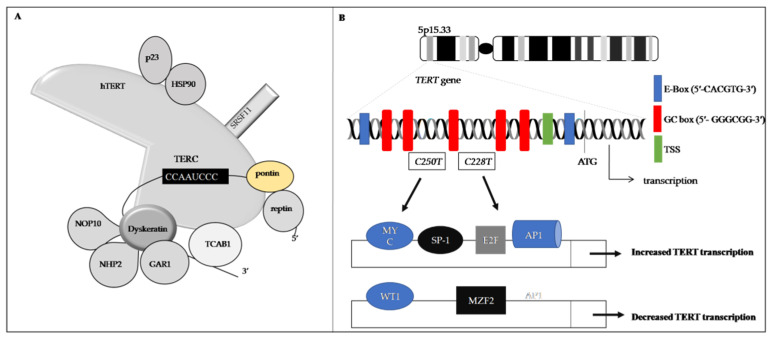
Schematic structure of telomerase. (**A**)Components of telomerase consist of the TERT (hTERT), the telomerase RNA template (TERC), and accessory proteins, including dyskerin, GAR1, NHP2, and NOP10. HSP 90, p23, pontin, reptin, and serine, SRSF11, and TCAB1. (**B**) The hTERT gene is situated on 5p15.33 which is responsible for 40 kb of human genome. The hTERT gene promoter contains five GC boxes (5′-GGGCGG-3′), two E-boxes (5′-CACGTG-3′), and one TSS. GC boxes are interacted with SP-1; E-boxes also have binding sites to MAD1; TSS binds THF1. Point mutations at the TERT promoter, predominantly at two points (C228T and C250T) generate new ETS/ternary complex (ETS/TCF) binding sites for transcription factors (TF). Increasing the expression of TFs such as c-MYC, ETS, NF-kB, and SP-1 results in binding to their particular sites and can up-regulate hTERT transcription. Binding of down-regulating transcription factors, such as WT1, CTCF, and MZF2, down-regulate TERT transcription. CTCF: CCCTC-binding factor; hTERT: human telomerase reverse transcriptase; NF-kB: nuclear factor kappa-light-chain-enhancer of activated B; TSS: transcription start site; WT1: Wilms tumor protein1; MZF2: myeloid zinc finger protein 2; GAR1: glycine-arginine rich 1; NHP2: non-histone protein 2; NOP10: nucleolar protein 10; HSP90: heat shock protein 90; TCAB1: telomerase Cajal body protein 1; SRSF11: serine and arginine-rich splicing factor 11.

Structurally, human telomerase consists of the TERT (hTERT), the telomerase RNA template (TERC), and accessory proteins. The telomerase catalytic protein component encoded by hTERT has telomerase activity, and this activity does not rely on the other components [3]. The hTERT can wrap the chromosome to add single-stranded telomere repeats [203]. The TERC contains the template for telomere replication [7]. The accessory proteins include: 1. Dyskerin, a highly conserved nucleolar protein that catalyzes the pseudouridylation of specific residues in newly synthesized ribosomal RNAs and spliceosomal small nuclear RNAs [204]. 2. Non-histone protein 2 (NHP2) and nucleolar protein 10 (NOP10), which are both ribonucleoproteins [205]. 3. Glycine-arginine rich 1 (GAR1), which is involved in RNA metabolism [206]. 4. p23, a small but important cochaperone for the Heat shock protein 90 (HSP90) chaperoning pathway, as part of the complex with telomerase [207]. 5. Telomerase Cajal body protein 1 (TCAB1) [13] is a telomerase holoenzyme and markedly enriched in Cajal bodies (CBs). In addition to regulating the subcellular location of telomerase [208], TCAB1 facilitates the recruitment of telomerase to CBs in the S phase of the cell cycle. This recruitment is dependent on TCAB1 binding to a telomerase RNA component [209]. 6. Reptin and pontin, two ATPases, interact with TERT in the S phase of the cell cycle [210].7. Serine and arginine-rich splicing factor 11 (SRSF11) is a TERC-binding protein that influences telomerase function, subcellular localization, and biogenesis [211]. Although hTERT and TERC are sufficient to reconstitute telomerase activity in vitro, a functional holoenzyme complex assembles hTERT, TERC, and accessory proteins in vivo to extend the telomere length in rapid cell divisions for attenuating or preventing telomere erosion [212] (Figure 4A). Furthermore, TERC is up-regulated in carcinomas affecting the cervix, head and neck, lung, and ovary—possibly serving as a therapeutic target [213,214].

### 5.2. TERT Is Important forthe Activity of Telomerase

In cancer cells, high telomerase activity breaks through the limitation of replication and avoids activation of the DNA damage signaling pathway [215]. Telomerase activity has been detected in 42–54% of thyroid cancer cases [216,217], 86.6% of non-small cell lung cancer cases [218], more than 80% of hepatocellular carcinoma cases [219], and 76% of cervical cancer cases [220]. These data suggest the criticality of the telomerase in cancer cells acquiring immortality or progression. While telomerase activation is poorly understood, hTERT is an important factor in telomerase activation. Generally, hTERT acts as the limiting factor for controlling telomerase activity and turns on the telomere clock for the aging process in all somatic adult cells [221]. Fibroblasts are a good example as fibroblasts do not express TERT and thus demonstrate gradual telomere shortening and eventual replicative senescence. However, introduced TERT expression in fibroblasts can maintain the telomere length and immortalize the cells [222]. Furthermore, hTERT is aberrantly expressed in approximately 90% of aggressive cancers [203,223,224] and 73% of tumor cases [225]. Therefore, increased TERT expression and telomerase activity can be detected in close to 90% of human cancers [226,227], highlighting the association of the hTERT with the telomerase in the emergence of malignant and aggressive phenotypes.

hTERT is situated on the 5p15.33 chromosome, which is responsible for 40 kb of the human genome [5,228]. hTERT has 16 exons and 15 introns that can produce 22 splicing variants and these variants can be dominant or negative [229]. The promoter responsible for hTERT transcription lacks typical regulatory sequences, such as the TATA and CAAT boxes, and instead comprises a transcription start site (TSS), two E-boxes (5′-CACGTG-3′), and five GC boxes (5′-GGGCGG-3′). TSS is a single transcription start site that multi-functional transcription factors such as THF1 can bind to. E-boxes have attachment sites for MAD1 to down-regulate the transcription of hTERT. GC boxes interact with the zinc finger transcription factor SP-1 [230]. The region (Figure 4B, white rectangles) represents a 260 bp hTERT core promoter region and has multiple binding motifs for enhancing factors, such as c-MYC, SP-1, E-twenty-six (ETS) family members, and NF-kB. hTERT transcription has also been reportedly down-regulated by the transcriptional factors CTCF, MZF-2, and WT1. Therefore, the hTERT promoter is pivotal for transcriptional activity [32] (Figure 4B).

### 5.3. Mechanisms Involved in TERT Activation

#### 5.3.1. TERT Promoter Is Critical in Cellular Immortality and Infinite Proliferation

In cancer cells, many mechanisms cause TERT activation. Several studies suggest that TERT is essential for malignant transformation, and transcriptional control of the TERT gene is highly regulated at various levels [231,232]. The mutation rate of the TERT promoter is more than 90% inhuman malignancies [232]. The mutation may activate telomerase, leading to infinite proliferation and infinite growth. Specifically, TERT promoter mutations may influence telomere length and affect gene expression [231]. TERT promoter mutations produce the ETS transcription factor family binding sites in multiple cancer types [232,233]. The ETS transcription factor family includes the activating GA Binding Protein Transcription Factor Subunit Alpha (GABPA) in a heterotetramer form with its counterpart, GA Binding Protein Transcription Factor Subunit Beta (GABPB), to activate TERT transcription and telomerase [233,234]. Consistently, thyroid carcinoma derived cells, which were knock-down GABPA, significantly down-regulated TERT expression [235] and GBM cells, which were knocked out of GABPB1, underwent apoptosis, and lost tumorigenic ability telomere shortening/dysfunction and proliferation/survival [234]. To sum up, inhibiting the GABPA or GABPB1 expression can lead to diminished TERT expression. These findings suggest that the TERT promoter mutations facilitate the binding of transcription factors, leading to the development of cancers.

#### 5.3.2. C228T and C250T: Gain-of-Function Mutation

TERT transcription can be activated by point mutations at the TERT promoter, predominantly at two points (C228T and C250T) [233]. Primary tumors bearing either mutation (C228T or C250T) tend to express higher levels of TERT mRNA and telomerase activity, implying a stimulatory effect on TERT expression [225,231]. Chiba et al. created a C228T mutation in the TERT promoter region in human iPSCs and found that these cells constitutively expressed TERT and telomerase even after having undergone terminal differentiation, in contrast to the wild-type TERT promoter-bearing stem cell-derived progenies, where the *TERT* transcription was shut down following cellular differentiation [236]. Li et al. introduced the C228T mutation into the TERT promoter in normal human bladder stem cells, and this single event was sufficient to drive the transformation of these stem cells [237]. Thus, these findings suggest that the presence of C228T or C250T mutation has a “gain-of-function effect” that can confer cell immortality, sustain proliferation potential, and promote cell transformation by activating TERT transcription. These mutations are first found in familial and sporadic malignant melanomas [229]. These mutations were then associated with enhanced telomerase activity in cancer cells [231]. This mutation pair results in TERT activation that then triggers the telomerase to elongate telomere length [184,238] and, as a consequence, leads to the immortal, anti-senescence, and proliferative properties of tumor cells. The C228T mutation is more readily detected in cancers than the C250T mutation [225,231]. The results of C228T and C250T may enhance GABPA or GABPB1 binding, inhibiting DICER1, leading to cell proliferation, immortality, and oncogenesis.

#### 5.3.3. Other Possible Mechanisms That Trigger TERT Activation

Several mechanisms, including TERT amplification, epigenetic changes [231], promoter methylation [239], alterations in alternative splicing of TERT pre-mRNA [214,228], chromatin remodeling, increased copy number, disruption of the telomere position effect (TPE) machinery [231], which is stronger with the longer telomere, and telomere shortening may lead to increased TERT transcription by the loss of TPE [240], which can trigger TERT activation. TPE has a silencing effect on genes located close to the telomeres [231]. TPE is also thought to be capable of regulating genes further away from the telomeres in what is called TPE over long distances (TPE-OLD) [232,241]. TERT expression is regulated by various transcription factors, such as MYC, SP-1, and E2F. AP-1, CCCTC binding factor, E2F, and estrogen response element (ERE) are associated with promoting TERT transcription [232,242,243]. Estrogen receptor α interacts with the TERT promoter and enhances the TERT mRNA output [232,242,243]. The phosphatidylinositol-3kinase/AKT pathway can phosphorylate TERT to increase the TERT functions [244,245].

*TERT* polymorphisms, such as rs2736100-CC genotype features, were reported more frequently than the wild-type TERT promoter in patients with cancer [246]. This genotype results in the elongation of telomeres through increasing TERT expression [247]. Although mammalian subtelomere regions are packed into constitutive heterochromatin, the detailed mechanisms of how epigenetic modifications impact telomere protection and structures are largely unknown. The heterochromatin is characterized by high levels of histone 3 lysine 9 trimethylation (H3K9me3), histone 4 at Lys20 (H4K20) trimethylation, and CpG methylation [248]. Stern et al. noted that a single nucleotide mutation in the TERT promoter led to the presence of the H3K4me2/3, which were associated with competent chromatin, and recruited the GABPA/B1 transcription factor that was transcriptionally active in several cancer cell lines [249].

#### 5.3.4. Telomeric Repeat-Containing RNA (TERRA)

Telomeric sequences contain TERRA, which is a very long, non-coding RNA [250]. TERRA can interact with TRF1 and TRF2 to regulate telomere length [250,251] and promote telomere shortening by inhibiting telomerase activity [252], promoting exonuclease 1 dependent resections [253], increasing the formation of euchromatin and decreasing heterochromatin formation [254]. The ability of TERRA to regulate telomere length is also related to human diseases. For instance, elevated levels of TERRA were found in ALT-positive tumors [255] and TERRA levels showed down-regulation following tumor grades in human astrocytoma cell lines [256]. These findings suggest that TERRA participates in the regulation of various physiological processes, such as telomerase activity, TERT and heterochromatinization, and disruptions to these processes can affect development and directly or indirectly induce disease. Moreover, TERRA is associated with telomeres via tethering in a DNA-RNA hybrid formation [257,258]. This structure may help to further understand the molecular interaction between DNA-RNA at the chromosome ends and to design specific telomere-targeting therapy in the future.

## 6. Telomerase-Based Anti-Cancer Strategy

The fundamental concept of cancer immunotherapy is based on manipulating the host immune system to attack the cancer cells. Although there are several novel cancer immunotherapy strategies, vaccine-based strategies are the most attractive and promising ones. However, it is very difficult to target tumor-associated antigens on the surface of tumor cells but not on that of the normal cells because of the heterogeneity and overlapping expression of these antigens in both cancers and healthy tissues [259]. As cancer cells lacking telomerase can undergo spontaneous remission, telomerase inhibition in most cancers may shed light on a potentially successful therapeutic strategy [260]. As telomerase is an HLA class-I antigen and can stimulate a cell-mediated immune response by inducing cytotoxic T-cells, numerous novel approaches have recently been developed to attenuate/inhibit the functions of the telomerase that impact cancer. Vaccination against telomerase is tolerable and safe and has been shown to induce excellent immunological responses associated with increased survival in several cancer types. Four well-known telomerase-based anticancer vaccines are discussed below.

### 6.1. GV1001

The GV1001, an HLA class II-restricted peptide vaccine, is composed of 16 amino acids (TERT_611–626_:EARPALLTSRLRFIPK) derived from the hTERT active site [32,261]. GV1001 was the first TERT peptide vaccine to be evaluated for treating advanced pancreatic cancer, lung carcinoma, melanoma, and liver carcinoma in clinical trials [261,262,263,264,265,266,267,268]. GM-CSF can enhance immunological response through the recruitment and maturation of dendritic cells and the activation of macrophages, neutrophils, and NK cells [269]; therefore, GV1001in combination with GM-CSF can result in a high frequency of immune responder s [32,262]. GV1001 can induce an efficient hTERT-specific T-cell activation and penetrate within tumor cells through the cell membrane [270]. Therefore, it can recognize the antigen-presenting cells that are internalized in the tumor and lymph nodes [262]. GV1001 can induce cancer cell apoptosis [271,272,273] and down-regulate heat shock proteins, hypoxia-inducible factor-1, and vascular endothelial growth factor to enhance its anti-tumor effect [270,272,274]. Although GV1001 is theoretically suitable for most cancers, a report suggests that the GV1001 vaccination is not effective in cutaneous T-cell lymphoma [275], and another report indicates that GV1001 cannot induce any specific immune responses in patients with advanced HCC [263], and the addition of GV1001 to chemotherapy (gemcitabine and capecitabine) did not show any significant clinical benefits [266]. Patients with tuberculosis or receiving tuberculin may not be suitable for GV1001 vaccination because the evoked immune response against mycobacterial peptides may be so dominant as to suppress the immune response against the hTERT peptide [264].

### 6.2. GX301 

The GX301 vaccine contains four immunogenic peptides (hTERT_540–548_: ILAKFLHWL; hTERT_611–626_: EARPALLTSRLRFIPK; hTERT_672–686_: RPGLLGASVLGLDDI, and hTERT_766–780_: LTDLQPYMRQFVAHL) that can bind both HLA class I and II; GX301 also contains two complementary adjuvants, Montanide ISA-51 and Imiquimod. Each GX301 administration consists of four intradermal injections (a fixed hTERT peptide dose, 500 µg)—one injection for each hTERT peptide—given at the same time and followed by topical application of imiquimod [276]. Montanide can protect the degradation of the peptides by tissue proteases, enhance peptide uptake by intradermal dendritic cells, induce interferon- γ release by innate immunity cells, and increase the expression of major histocompatibility complex (MHC) by tumor cells [277]. Imiquimod can activate the Toll-like receptor-7 and receptor-8 and induce the activation and maturation of dendritic cells [278]. The immunogenicity of GX301 was demonstrated in an ex vivo study in which circulating T-cell responses to its hTERT peptides were detected in all subjects [276]. A phase I trial of GX301 has provided evidence of vaccine-specific immune response in patients with stage IV prostate and kidney cancer, and prolonged progression-free survival and overall survival were observed in patients showing a full pattern of vaccine-specific immunologic responses [276]. A phase II, randomized, parallel-group, open-label, multicenter trial (EudraCT: 2014-000095-26 and ClinicalTrials.gov Identifier: NCT02293707) has demonstrated that all the patients showed good immune responses to at least one of the peptides. The overall response was more for the multi-peptide vaccines than the single-peptide vaccines [279], suggesting that the four GX301 peptides endow a cumulative epitope pattern wide enough for inducing telomerase-specific peripheral T-cell reactivity in most individuals. A phase II, multicenter, randomized, parallel-group, open-label trial (EudraCT:2014-000095-26 and ClinicalTrials.gov Identifier:NCT02293707) was designed to comparatively analyze the safety and immunological response to GX301 regimens in castration-resistant prostate cancer patients with response/disease stability after docetaxel chemotherapy. Although the results indicate that the GX301 cancer vaccine is safe and 95% of the patients showed at least one vaccine-specific immune response, the overall survival did not differ between immunological responders and non-responders [280].

### 6.3. UV1

UV1 is a second-generation, multi-peptide vaccine constituted by three hTERT-derived peptides (hTERT_652–665_: AERLTSRVKALFSVL; hTERT_660–689_: ALFSVLNYERARRPGLLGASVLGLDDIHRA and hTERT_691–705_: RTFVLRVRAQDPPPE) [262]. In phase I and IIa trials, UV1 was administered along with GM-CSF for six months in patients with metastatic prostate cancer in combination with radiotherapy and androgen deprivation treatment (ADT). A total of 85.7% of patients showed an immune activation and 64% showed reduced levels of the prostate-specific antigen (PSA). In addition, 45% of the patients showed no evidence of the disease at the end of the trial [281]. Several checkpoint inhibitors, including Ipilimumab (anti-CTLA-4) or pembrolizumab (anti-PD-1) in melanoma patients (NCT02275416 and NCT03538314, respectively) and ipilimumab in association with nivolumab (anti-PD-L1) in patients affected by mesothelioma (NIPU trial, NCT04300244) have been singly or multiply used in combination with UV1 in clinical trials. The results showed that the treatment of UV1 together with these checkpoint inhibitors were safe and well-tolerated, and no severe allergic reactions were observed [282,283,284]. The NIPU trial is still ongoing and the primary end-point is expected to be analyzed in 2022.

### 6.4. Vx-001

Vx-001 is a peptide-based cancer vaccine consisting of two peptides: hTERT-derived low-affinity cryptic hTERT peptide: TERT 572 (RLFFYRKSV; ARG-Vx001) and its optimized mutant hTERT peptide: TERT 572Y (YLFFYRKSV; TYR-Vx001), which has an enhanced affinity to MHC class I molecules as the first amino acid was replaced with a tyrosine residue [285]. The antitumor efficacy and safety of Vx-001 has also been investigated in phase I/II clinical trials for different cancers, such as melanoma, bile duct cancer, breast cancer, and lung cancer. Results of these trials show that Vx-001may elicit a specific and possibly optimal cytotoxic T cell response against hTERT-expressing tumor cells and has improved clinical outcomes in clinical trials without any relevant toxicity [286,287,288,289].

Collectively, the hTERT-vaccine clinical trials indicate that these immunotherapies may represent a promising approach in cancer treatment. Apart from the TERT peptide vaccines, several novel immunotherapies, including the dendritic cell-based tumor vaccine, such as GRNVAC1 [289] and GRNVAC2 [290,291]; Tumor Antigen Presenting Cells (TAPCells) vaccines [292]; DNA vaccines such as phTERT [293], INVAC-1 [294]; adenovirus type 6 of an anticancer vaccine expressing hTERT, such astheV934/V935 vaccine [295]; gene-modified T-cell therapy, such as the use of tumor antigen-specific T-cell receptors originating from tumor-specific T cells or their clones [296,297]; the use of a chimeric antigen receptor (CAR) [298,299]; the molecules inhibiting Ras farnesylation [76], and hTERT-expressing human umbilical endothelial cells (HUVEC-TERTs) [300], may be effective without prominent toxicity.

## 7. Alternative Lengthening of Telomere (ALT)

### 7.1. ATRX and DAXX

Even with these new therapies, there have been certain cancers that can evade treatment by using an alternative lengthening of the telomere (ALT) mechanism. ALT is a telomerase-independent mechanism that uses recombination-dependent pathways to increase telomere length [301]. ALT is present in non-neoplastic tissues and in stromal, endothelial, and epithelial cells [302] and in approximately 10-15% of cancers [303], and it is common in sarcoma and glioma [304,305]. In the absence of telomerase, the ALT pathway uses a homologous recombination-based DNA replication mechanism to gain immortality. ALT activation required two chromatin-remodeling factors: the α-thalassemia X-linked intellectual disability (ATRX) and the death domain-associated protein (DAXX) [304,306]. DAXX was initially describe as a Fas death receptor binding protein [307]. ATRX is widely expressed and is a multi-functional factor involved in chromatin organization, DNA methylation, and transcriptional regulation [308]. Mutations in ATRX result in α-thalassemia ATRX syndrome, which is characterized by severe developmental delays, peculiar facial hypotonia and a characteristic mouth, intellectual impairment, genital anomalies ranging from undescended testes to ambiguous genitalia, and anemia secondary to α-thalassemia [309]. Patients with this syndrome may present long telomeres, which may be due to either improper maintenance of telomeric heterochromatin, improper resolution of replication stress at telomeres, or both by the mutation of ATRX [310].

### 7.2. Correlation between the Loss-of-Function of ATRX/DAXX and ALT in Cancer

The mutated *ATRX* gene is frequently detected in several tumors, including adrenocortical carcinoma, gliomas, GBM, neuroblastoma, and osteosarcoma [306], and pancreatic neuroendocrine tumors(panNETs), which are a group of endocrine tumors arising in the pancreas. PanNETs are among the most common neuroendocrine tumors. Functioning panNETs include insulinoma, gastrinoma, vasoactive intestinal peptide tumors (VIPoma), glucagonoma, and others that produce specific hormonal hypersecretion syndromes. Endocrine testing, imaging, and histological evidence is necessary to accurately diagnose panNETs. PanNETs may or may not cause signs or symptoms; however, as most panNETs may have malignant potential, an aggressive therapeutic approach for panNETs, including surgery, locoregional therapy, systemic therapy, and complication control, is required [311]. A report showed that 43% of panNETs contained the mutated *ATRX* or *DAXX* [312]. A correlation between the loss-of-function of ATRX/DAXX and the ALT phenotype in panNETs was found [313] and ATRX was proposed to serve as the primary suppressor of ALT [314]. Furthermore, when ATRX was reintroduced into ALT-positive ATRX-negative cell lines it was found to eliminate ALT-associated phenotypes [315,316]. Gliomas with wild-typeTERT promoters often present ATRX mutations to activate ALT [317]. A fibrosarcoma cell line (HTC75), which is telomerase-positive, can be converted to an ALT-mediated telomere elongation mechanism through TERT knockout, and the subsequent changes result in telomeric DNA damage and disruption of the ATRX/DAXX complex, indicating a negative correlation between mutations affecting TERT and ATRX/DAXX [304]. Consequently, telomeric DNA damage can reduce the compaction of telomeric chromatin, resulting in the production of altered telomeric DNA sequences. This in turn activates a telomere-specific DDR pathway [12,316], which can stimulate the homology-directed synthesis of telomeric DNA. However, cancer cells can circumvent cell death caused by an absence of telomerase or dysfunction by switching from telomerase-dependent to ALT-mediated telomere lengthening [318,319].

### 7.3. Targeting Telomerase Activity and the ATRX/DAXX Complex

Direct and indirect approaches to targeting telomerase activity and the ATRX/DAXX complex could prove effective. Direct approaches include immunotherapy specifically targeting TERT tumor-associated antigens, such as anti-sense oligonucleotides (e.g., Imetelstat/GRN163L) and small-molecule inhibitors (e.g., BIBR1532) and small molecule inhibitors could be used to bind telomerase and inhibit telomere elongation. Indirect techniques, such as G-quadruplex stabilizers (e.g., RHPS4, Telomestatin, TMPyP4, CX-3543/quarfloxacin), which are designed to block telomerase activity, are promising [320]. The G-rich oligos, which homolog to the telomeric overhang that forms the G4 structures, cause telomere dysregulation and a decreased proliferation rate, enhance apoptosis, and reduce expression of the TERT within melanoma cells [321].An alternative approach is based on telomere uncapping, using nucleoside analogs(e.g., 6-thio-dG) that rapidly affect telomere dysfunction, quickly triggering cancer cell death [322]. In addition, other factors, such as transcriptional, posttranscriptional, and epigenetic modifications can affect the activation or silencing of TERT; however, the effects are poorly understood in somatic, cancer, and stem cells. Epigenetic regulators, such as non-coding RNAs, histone modification, and DNA methylation, are now seen as crucial components for the regulation of telomeres and telomerase activity [323] and unlocking the epigenetic mechanisms associated with telomerase regulation could see advances in cancer diagnosis, treatment, and prognosis [324]. Convergently, a multipronged treatment strategy can maximize anti-tumor effects.

## 8. Conclusions

Telomeres are hexameric DNA repeats situated at the ends of human chromosomes and are associated with genome replication and protection. Telomere length regulation has been attributed to several functional modulators, telomerase, and two important protein complexes, shelterin and CST synergizing with DNA replication, repair, and RNA metabolism pathway components. The mechanisms involving telomere maintenance play a critical role in cancer development and thus form the primary targets for the development of cancer therapeutics. In the majority of cancers, tumors attain replicative immortality primarily through telomerase activation via increased TERT transcription, providing telomerase as the preferred target for drug development in cancer therapeutics.

Among numerous novel anticancer therapies, vaccine-based strategies are the most attractive and promising approach. While there is much excitement in the telomerase-against-cancer arena, many challenges and questions still remain unanswered. Although much work is needed, several clinical trials have shown the effective anticancer responses of these telomerase-based anticancer vaccines without toxicity to non-cancer cells. Furthermore, the suppression of CST, shelterin, and ALT cells may inhibit cell proliferation. Combining vaccines with the targeted manipulation of the immune response through different pharmacological approaches may improve the overall efficacy of telomerase-based anti-cancer immunotherapies in future trials.

## Figures and Tables

**Table 1:** Comparison of CST and RPA.

CST.	Component	aa	OB	wH	wHTH1	Functions	References
						Binding to ssDNA.	[119,133,134,135,136,137]
	CTC1	1217	7	0	0	2. Binding to ssDNA-dsDNA junctions.	[138]
	STN1	368	1	0	2	3. Recognize different specialized DNA structures at DNA replication and breakage sites.	[139]
	TEN1	123	1	0	0	4. Acting synergistically with ATR to maintain telomere length and genome stability.	[140]
						5. Stimulating Polα.	[141,142,143]
						6. Helping in C-strand fill-in.	[136,144]
						7. Preventing the accumulation of G4.	[139]
						8. Preventing telomeric DNA damage.	[125,134]
						9. Interacting with the MCM and disrupting binding of CDT1 to MCM, leading to decreased origin licensing.	[145]
						10. Interacting with AND-1.	[146]
						11. Inhibiting telomerase.	[138,143,144,147,148]
RPA						1. Binding ssDNA.	[149]
	RPA70	616	4	0	0	2. Activating the ATR signaling.	[150]
	RPA32	270	1	1	0	3. Activating the helicase.	[151]
	RPA14	121	1	0	0	4. Unwinding G4.	[152]
						5. Involved in DNA replication, recombination, and repair.	[62]
						6. Activating BLM’s bidirectional DNA unwinding.	[153]
						7. Modulating the fork remodeling enzyme activity.	[154]
						8. Enhancing primase.	[155]

## Data Availability

Not applicable because this review did not report any data.
